# Non-invasive Brain Stimulation in the Treatment of Post-stroke and Neurodegenerative Aphasia: Parallels, Differences, and Lessons Learned

**DOI:** 10.3389/fnhum.2016.00675

**Published:** 2017-01-23

**Authors:** Catherine Norise, Roy H. Hamilton

**Affiliations:** Laboratory for Cognition and Neural Stimulation, Department of Neurology, University of PennsylvaniaPhiladelphia, PA, USA

**Keywords:** aphasia, primary progressive aphasia, stroke, tDCS, neurorehabilitation

## Abstract

Numerous studies over the span of more than a decade have shown that non-invasive brain stimulation (NIBS) techniques, namely transcranial magnetic stimulation (TMS) and transcranial direct current stimulation (tDCS), can facilitate language recovery for patients who have suffered from aphasia due to stroke. While stroke is the most common etiology of aphasia, neurodegenerative causes of language impairment—collectively termed primary progressive aphasia (PPA)—are increasingly being recognized as important clinical phenotypes in dementia. Very limited data now suggest that (NIBS) may have some benefit in treating PPAs. However, before applying the same approaches to patients with PPA as have previously been pursued in patients with post-stroke aphasia, it will be important for investigators to consider key similarities and differences between these aphasia etiologies that is likely to inform successful approaches to stimulation. While both post-stroke aphasia and the PPAs have clear overlaps in their clinical phenomenology, the mechanisms of injury and theorized neuroplastic changes associated with the two etiologies are notably different. Importantly, theories of plasticity in post-stroke aphasia are largely predicated on the notion that regions of the brain that had previously been uninvolved in language processing may take on new compensatory roles. PPAs, however, are characterized by slow distributed degeneration of cellular units within the language system; compensatory recruitment of brain regions to subserve language is not currently understood to be an important aspect of the condition. This review will survey differences in the mechanisms of language representation between the two etiologies of aphasia and evaluate properties that may define and limit the success of different neuromodulation approaches for these two disorders.

## Introduction

In recent years there has been a surge of interest in the application of non-invasive brain stimulation (NIBS) techniques such a transcranial magnetic stimulation (TMS) and transcranial direct current stimulation (tDCS) to the treatment of a variety of conditions in psychiatry, neurology, and rehabilitation. In part, this move toward exploring the use of non-invasive neuomodulation is fueled by an increasing understanding of the mechanisms that drive abnormal structural and functional organization of the brain in psychiatric and neurologic disease, and an increasing realization that improved characterization of these mechanisms affords greater opportunities for focused intervention. Clinically-oriented discoveries in systems-level neuroscience have led to a sea change in the conceptualization of a variety of disease processes such as depression (Boggio et al., 2008; Brunoni et al., 2011, 2012), dementia syndromes (Freitas et al., 2011; Elder and Taylor, 2014), traumatic brain injury (Demirtas-Tatlidede et al., 2012), and focal deficits after stroke (Schjetnan and Escobar, 2012; Blesneag et al., 2015), not simply as problems with specific regions of the brain, but as problems with brain networks, the treatment of which is directly linked to the ability to harness and modulate the brains' capacity for functional reorganization.

Evidence indicates that recovery of language abilities in patients with aphasia depends on reorganization of brain function (Saur et al., 2006; Sarasso et al., 2010; Abel et al., 2015). Recent advancements in our understanding of the neurobiology of language and cognitive neuroscience has revealed the importance of dynamic alteration of the normally left-hemisphere dominant language network in the setting of aphasia and aphasia recovery (Fridriksson, 2010; Hamilton et al., 2011; Fridriksson et al., 2013). For instance, a recent a study by Fridriksson and colleagues indicated that alterations in activation of perilesional areas could predict treatment response in patients with chronic aphasia (Fridriksson et al., 2013).

Aphasia is most commonly seen after stroke, however a second pathologic process that can commonly lead to deficits of language is neurodegenerative disease. As the population ages neurodegenerative disorders represent a growing epidemic. Neurodegenerative dementias like Alzheimer's disease and frontotemporal degeneration frequently manifest with language disorder syndromes, which are collectively referred to as primary progressive aphasias (Mesulam, 2001).

There are a number of important phenomenological overlaps between aphasia due to stroke and primary progressive aphasia (PPA). There are also a number of notable differences. Undoubtedly, some of the key differences are mediated by a critical distinction in the underlying pathological processes between the two conditions: Aphasia after stroke is the result of acute, focal obliteration of components of the brain's language network, whereas PPA can be conceptualized as a gradual progressive degradation of the efficiency and activity of this network. This difference is likely to lead to important further distinctions in whether and how the representation of the language in the brain changes over time in these two disease processes. Examination of this topic may reveal important insights into how mechanisms of neurologic disease can be used to guide neuromodulatory therapies. At the same time, exploring the effects of brain stimulation on two different mechanisms of aphasia may also further inform our understanding of language systems in the brain, and the compensatory neuroplastic processes employed by neural systems to mediate recovery from aphasia (Tables 1, 2).

**Table 1:** **Inclusion exclusion criteria for identifying treatment studies**.

	**Inclusion Criteria for identifying treatment studies included in this review**	**Exclusion criteria for identifying treatment studies included in this review**
Patients	Studies that included adults diagnosed with primary progressive aphasia or aphasia due to stroke No restrictions were applied based on the: ° Type of stroke (ischemic or hemorrhagic) ° Recovery phases (acute, sub-acute, and chronic) ° Specific anatomical location of lesion and/or atrophy ° Disease severity	Studies that included patients who did not suffer from aphasia (e.g., Alzheimer's disease, Parkinson's) Non-human subjects
Treatment	Studies that included rTMS or tDCS as treatment No restriction on the site of stimulation were applied No restriction as to the specific brain stimulation paradigms were applied: ° For tDCS, studies that applied anodal or cathodal tDCS, or both ° For TMS, studies that applied repetitive TMS (low or high frequency) No restriction on the duration or timing of SLT (offline or online)	Studies that included rTMS or tDCS but not as a treatment Speech intervention studies such as melodic intonation therapies, but without rTMS or tDCS
Trial designs	Between-subject, randomized controlled trials, cross-over trials, case reports, within subject or pre-post trial designs	Review articles and book chapters

**Table 2:** **Included treatments studies**.

**Authors**	**Diagnosis**	**NIBS**	**Frequency/Orientation**	**Sample size**	**Chronicity**	**Control**
Tsapkini et al., 2014	PPA–non-specified	tDCS	L-anodal	6		Sham (within subject crossover)
Cotelli et al., 2014	naPPA	tDCS	L-anodal	16		Sham controlled (*n* = 8)
Wang et al., 2013	naPPA	tDCS	L-anodal	1		Sham (within subject crossover)
Finocchiaro et al., 2006	PPA–non-specified	TMS	L-high frequency	1		Sham controlled
Cotelli et al., 2012	naPPA an svPPA	TMS	L-high frequency, R-high frequency	10		Sham controlled
Trebbastoni et al., 2013	lvPPA	TMS	L-high frequency	1		Sham (within subject crossover)
Galletta and Vogel-Eyny, 2015	Post-stoke non-fluent aphasia	tDCS	L-anodal	1	Chronic	Sham (within subject crossover)
Shah-Basak et al., 2015	Post-stoke non-fluent aphasia	tDCS	L-anodal, L cathodal, R-anodal, R-cathodal	7	Chronic	Sham partial crossover (*n* = 3)
Wu et al., 2015	Post-stoke non-fluent aphasia	tDCS	L-anodal	12	Chronic (*n* = 4); Subacute (*n* = 8)	Sham controlled; and healthy controls (*n* = 12)
Vestito et al., 2014	Post-stoke non-fluent aphasia	tDCS	L-anodal	3	Chronic	Sham controlled
Lee et al., 2013	Post-stoke non-fluent aphasia	tDCS	L-anodal and R-cathodal	11	Chronic	Crossover between single and dual electrode stimulation
Marangolo et al., 2013	Post-stoke non-fluent aphasia	tDCS	L-anodal and R-anodal	12	Chronic	Sham controlled; and healthy controls (*n* = 20)
Marangolo et al., 2011	Post-stoke non-fluent aphasia	tDCS	L-anodal	3	Chronic	Sham (within subject crossover)
Datta et al., 2011	Post-stoke non-fluent aphasia	tDCS	L-anodal	1	Chronic	Sham controlled
You et al., 2011	Post-stoke non-fluent aphasia	tDCS	L-anodal, R-cathodal	78	Chronic	Sham controlled
Fridriksson et al., 2011	Post-stoke non-fluent aphasia	tDCS	L-anodal	8	Chronic	Sham controlled
Fiori et al., 2011	Post-stoke non-fluent aphasia	tDCS	L-anodal	3	Chronic	Sham controlled; and healthy controls (*n* = 10)
Baker et al., 2010	Post-stoke non-fluent aphasia	tDCS	L-anodal	10	Chronic	Sham controlled
Flöel et al., 2011	Post-stoke non-fluent aphasia	tDCS	R-anodal, R-cathodal	12	Chronic	Sham controlled
Monti et al., 2008	Post-stoke non-fluent aphasia	tDCS	L-anodal and L-cathodal	8	Chronic	Sham controlled
Dammekens et al., 2014	Post-stoke non-fluent aphasia	TMS	L-high frequency	1	Chronic	Sham (within subject crossover)
Al-Janabi et al., 2014	Post-stoke non-fluent aphasia	TMS	R-high frequency	2	Chronic	Sham controlled
Rosso et al., 2014	Post-stoke non-fluent aphasia	tDCS	R-cathodal	25	Chronic	Sham controlled
Santos et al., 2013	Post-stoke non-fluent aphasia	tDCS	R-cathodal	19	Chronic	No control
Jung et al., 2011	Post-stoke non-fluent aphasia	tDCS	R-cathodal	37	Subacute and Chronic	No control
Kang et al., 2011	Post-stoke non-fluent aphasia	tDCS	R-cathodal	10	Chronic	Sham (within subject crossover)
Martin et al., 2014	Post-stoke non-fluent aphasia	TMS	R-low frequency	2	Chronic	Within subject control
Heiss et al., 2013	Post-stoke non-fluent aphasia	TMS	R-low frequency	29	Subacute	Sham controlled
Medina et al., 2012	Post-stoke non-fluent aphasia	TMS	R-low frequency	10	Chronic	Sham partial crossover (*n* = 5)
Naeser et al., 2012	Post-stoke non-fluent aphasia	TMS	R-low frequency	2	Chronic	Sham controlled
Barwood et al., 2012	Post-stoke non-fluent aphasia	TMS	R-low frequency	7	Chronic	No control
Naeser et al., 2011	Post-stoke non-fluent aphasia	TMS	R-low frequency	8	Chronic	Healthy controls (*n* = 8)
Barwood et al., 2011	Post-stoke non-fluent aphasia	TMS	R-low frequency	12	Chronic	Sham controlled (*n* = 6)
Martin et al., 2009	Post-stoke non-fluent aphasia	TMS	R-low frequency	2	Chronic	No control
Costa et al., 2015	Post-stoke non-fluent aphasia	tdcs	Bihemisphere: L-anodal and R-cathodal	1	Chronic	Sham controlled
Marangolo et al., 2014	Post-stoke non-fluent aphasia	tdcs	Bihemisphere: L-anodal and R-cathodal	7	Chronic	Sham controlled
Khedr et al., 2014	Post-stoke non-fluent aphasia	TMS	L-high frequency and R-low frequency	30	Subacute	Sham controlled
Winhuisen et al., 2005	Post-stoke non-fluent aphasia	TMS	L and R low frequency	11	Subacute	Healthy controls

*PPA, primary progrssive aphasia; naPPA, non-fluent aggramatic variant PPA; svPPA, semantic varient PPA; lvPPA, logopenic PPA; NIBS, non-invasive brain stimulation, Subacute < 6 month post-stroke, Chronic > 6 months post-stroke*.

## Comparing causes and consequences of aphasia

Aphasia, is one of the most common post-stroke cognitive disorders. Stroke affects 795,000 Americans, and there are approximately 80,000 new cases of aphasia per year (Wade et al., 1986; National Stroke Association, 2008; Kyrozis et al., 2009; American Heart Association, 2016). Aphasia in the setting of stroke most often develops after left middle cerebral artery (Warburton et al., 1999) or left internal carotid artery territory infarcts. Depending on the location of the lesion in stroke, the clinical presentation of aphasia can be characterized by different profiles of language deficits.

The clinical characterization of aphasia syndromes due to stroke has been dominated by a model that was built and refined by critical clinical observations of Broca, Werinicke, Lichtheim, and later Geschwind (Broca, 1861; Wernicke, 1874; Geschwind, 1972; Prins and Bastiaanse, 2006). However, recent advances in the neurobiology of language have expanded aspects of this classical model in a variety of ways that are beyond the scope of this review (Poeppel, 2012). Therefore, without diminishing the historical importance of this model, which remains highly clinically relevant, we will largely limit our discussion of aphasia presentations to the symptom elements that have been used to define classic aphasia syndromes: fluency, comprehension, repetition, and naming ability.

Impaired fluency has been associated with lesions to the left inferior frontal gyrus, including Broca's area, but are also observed with lesions to the putamen or the anterior centrum semiovale (Kreisler et al., 2000). Patients with non-fluent aphasia due to stroke commonly exhibit effortful speech characterized by the inability to formulate grammatically correct utterances (agrammatism). In classical post-stroke aphasia models, deficits in the comprehension of words and sentences traditionally localize to injury of the posterior aspect of the superior temporal gyrus (Wernicke's area), as well as surrounding cortical areas and underlying white matter structures (Alexander, 1997). When more anterior regions of the perisylvian cortex are spared, these patients are left with a fluent aphasia in which the rate and flow of words is relatively preserved, despite impairment in the communication of content. Of note, errors of grammatical comprehension can also be seen with lesions of the inferior frontal gyrus, reflecting the frontal lobe's broader role in organizing grammatical constructions (Peelle et al., 2008; Charles et al., 2014). Relatively isolated deficits in repetition—a finding seen in so-called conduction aphasia—have traditionally been localized to lesions of the arcuate faciculus, but are also seen with damage to other areas including the left superior marginal gyrus and sometimes extend to the temporal cortex (Kreisler et al., 2000; Bernal and Ardila, 2009; Yourganov et al., 2015). Naming localizes poorly in the dominant hemisphere and is associated with lesions throughout the perisylvian cortex, underlying white matter pathways, and even deep gray areas like the thalamus (Kreisler et al., 2000). The degree to which individuals recover from post-stroke aphasia is variable (Laska et al., 2001; Meinzer et al., 2010), and persistent deficits are common (Robey, 1994; Robey and Wambaugh, 1999; Basso and Marangolo, 2000; Nickels, 2002). Unfortunately, despite continuing advances in our understanding of the neurobiology and cognitive neuroscience of language and aphasia, the efficacy of current behaviorally-based rehabilitation approaches remains quite limited (Mimura et al., 1998; Rosen et al., 2000; Winhuisen et al., 2005; Heiss and Thiel, 2006; Saur et al., 2006).

Primary progressive aphasia (PPA) refers to acquired language impairments that result from neurodegenerative diseases that affect cognition. Thus, far three variants of PPA have been identified: non-fluent, semantic, and logopenic. Similar to the deficits observed in aphasias due to stroke, those in PPA are characterized by impairments in fluency, comprehension, repetition, and naming. Each variant of PPA is characterized by a separate pattern of atrophy and a distinct clinical presentation. In the non-fluent variant of PPA, atrophy is typically observed in the left posterior frontal and insular region, including the inferior frontal gyrus, which overlaps to a large degree with the area of injury often seen in patients with non-fluent aphasia due to stoke. In some cases, atrophy is also distributed across the insula, and is seen in premotor and supplementary motor areas (Josephs et al., 2008; Gorno-Tempini et al., 2011; Wilson et al., 2011). The typical clinical presentation for these patients, like that seen in patients with non-fluent aphasia due to stroke, involves effortful speech, agrammatism, and production errors (Mesulam, 2001, 2008; Gorno-Tempini et al., 2011).

Clinically, semantic variant PPA shares some features with Wernicke's aphasia in that it is characterized by impairments in naming and single-word comprehension (Hodges and Patterson, 2007; Gorno-Tempini et al., 2011; Thompson et al., 2015). However, a notable difference between the two clinical presentations is that while repetition is impaired in Wernicke's aphasia, it can be relatively spared in the semantic variant of PPA (Hodges and Patterson, 2007; Gorno-Tempini et al., 2011; Thompson et al., 2015). Patterns of neural injury also differ between the two, in that the semantic variant of PPA features atrophy predominantly affecting the anterior, ventral, and lateral aspects temporal lobes. By contrast, the lesioned area in Wernicke's aphasia is traditionally seen in the posterior aspect of the left temporal lobe, although some recent evidence suggests that the anterior temporal lobe may also play an important role in language comprehension (Binder, 2015). While temporal lobe atrophy is often seen bilaterally in semantic variant PPA, it is typically most pronounced on the left (Hodges and Patterson, 2007).

Lastly, the logopenic variant of PPA, shares some similarities with conduction aphasia, in that it is associated with cerebral atrophy involving the posterior superior temporal gyrus, supramarginal gyrus, and angular gyrus, which overlaps with regions associated with conduction aphasia in patients with stroke. Reminiscent of conduction aphasia associated with stroke, one of the prominent features observed in the logopenic variant of PPA is impairment of repetition. However, logopenic PPA is also characterized by several other deficits, including impaired single word retrieval and phonologic errors in spontaneous speech (Gorno-Tempini et al., 2011). The term “logopenic” translates to “few words,” and in keeping with that label, the speech of patients with this variant of PPA is slow and halting with frequent pauses due to word-finding difficulties. However, unlike the non-fluent variant of PPA or most patients with non-fluent aphasia due to stroke, grammar is generally preserved in patients with the logopenic variant.

In summary, while the underlying pathoetiology clearly differs between these two causes of aphasia–lesion vs. atrophy—the three variants of primary progressive aphasia share some of the clinical deficits and anatomic regions of damage with the aphasia syndromes seen in aphasias after stroke including non-fluent aphasias (e.g., Broca's aphasia), fluent aphasias (e.g., Wernicke's aphasia), and conduction aphasia. Mounting evidence supports neuromodulation as a therapeutic option for language recovery in aphasia. As we will discuss below, there is now substantive evidence supporting the effectiveness of brain stimulation in treating aphasia due to stroke, and a small but growing body of evidence demonstrating the promise of these technologies in treating patients with PPA.

## Non-invasive brain stimulation in the treatment of aphasia

To date, two forms of NIBS have been explored as potential treatments for aphasia: (TMS) and (tDCS). TMS creates a fluxing magnetic field, which allows for the generation of current in underlying cortical neurons, causing them to depolarize. As a result, TMS can be used to manipulate cortical function in a focal manner, effectively allowing for investigation of structure-function relationships in the human cerebral cortex (Pascual-Leone et al., 1998). tDCS, another form of NIBS that has been employed in aphasia, modulates brain activity by delivering a weak polarizing electrical current, which subtly but demonstrably modulates neural activity in the cortex (Schlaug et al., 2009). In addition to the mechanistic differences between tDCS and TMS, these two technologies also differ in several practical ways that are germane to their potential use as therapies. tDCS is a less expensive and much more portable option compared to TMS. The sham condition in tDCS is more reliable, as patients are often able to perceive the sensation of the stimulus in TMS (Priori et al., 2009; Ambrus et al., 2012). The two methods also differ with regards to spatial resolution, where TMS provides focal stimulation and conventional tDCS provides a more distributed current flow in the brain, although recently developed “high-definition” tDCS systems may allow for more focused electrical stimulation (Datta et al., 2009). Lastly the manner in which the current is delivered in tDCS—that is, electrodes strapped to the scalp—allows for greater freedom of movement such that tDCS can be paired with other treatments like physical or speech therapy compared to TMS (Priori et al., 2009).

Non-invasive brain stimulation (NIBS) techniques have been shown to facilitate neuroplastic changes and to yield cumulative effects that last beyond the time of stimulation (Pascual-Leone et al., 1994; Bolognini et al., 2009; Rossi et al., 2009). The biological underpinnings of stimulation-induced changes in neural function are not completely understood. A recent review by Bolognini et al. (2009) highlights evidence that suggests that long-term changes in neural activity and behavior related to TMS are mediated by well-known neural mechanisms of plasticity, including long-term potentiation (LTP) or long-term depression (LTD) seen following repetitive activation of synaptic pathways (Hoffman and Cavus, 2002; Huang et al., 2005), sustained modulation of neurotransmitter levels (Hasselmo, 1995; Strafella et al., 2001, 2003), and gene induction (Hausmann et al., 2000). The same review also explored potential underlying mechanisms of tDCS, suggesting that the long-term effects that accompany this form of stimulation may also be due to synaptic mechanism similar to LTP and LTD (Bolognini et al., 2009). Additionally, the glutamatergic system, specifically NMDA, has been show to play a role in the maintenance of neuroplastic change following stimulation (Liebetanz et al., 2002; Paulus, 2004).

While the various mechanisms described above provide plausible explanations the behavioral effects of neuromodulation, it is noteworthy that the reliability and to some extent the efficacy of brain stimulation techniques—particularly tDCS—remains controversial. In different quantitative reviews and meta-analyses, investigators have debated whether the effects of tDCS on cognition and neurophysiology are reliable, consistent, and predictable (e.g., Horvath et al., 2015, 2016, but also Price et al., 2015). This ongoing controversy underscores the importance of conducting well-controlled, adequately powered studies employing brain stimulation, including studies of aphasia. Importantly, with respect to patients with aphasia, the notion that brain stimulation results can be unreliable or inconsistent further emphasizes the importance of characterizing underlying mechanisms of language reorganization in both post-stroke and neurodegenerative aphasia, and of understanding how these mechanisms ought to inform brain stimulation approaches.

## TMS and tDCS in the treatment of post-stroke aphasia

### TMS

To date, most of the TMS research involving brain stimulation and aphasia has been conducted on post-stroke aphasic patients. These studies frequently involve low frequency (1–4 Hz) stimulation to the right hemisphere at sites that are homotopic to damaged left hemisphere structures that are generally understood to be components of the intact language network. The stimulation typically lasts for 20–40 min per session over the course of 10–15 days, and the demonstrated language improvements have, in a number of studies, been shown to persist for months after the discontinuation of stimulation (Naeser et al., 2005; Barwood et al., 2011, 2013; Weiduschat et al., 2011; Abo et al., 2012; Medina et al., 2012; Waldowski et al., 2012; Thiel et al., 2013). For instance, in a case-study reported by our lab, we demonstrated that when low-frequency TMS was applied to the inferior frontal gyrus of the intact right hemisphere of a patient with chronic non-fluent aphasia, there were marked improvements in object naming and spontaneous speech that were maintained for 10 months following a 10-day course of stimulation (Hamilton et al., 2010).

A recent meta-analysis explored the utility of low-frequency repetitive transcranial magnetic stimulation (rTMS). Across 4 articles and 132 patients inhibitory TMS facilitated improvements in naming more so than repetition or comprehension (Li et al., 2015). A second meta-analysis focused specifically on studies that applied low-frequency TMS to the right inferior frontal gyrus of patients with subacute and chronic aphasia after stroke. In this study, Ren and colleagues demonstrated that inhibitory TMS to homotopic areas in the right hemisphere enhanced language recovery, as measured by aphasia severity, expressive language, and receptive language (Ren et al., 2014).

There are different theoretical models that account for how the brains of patients with stroke reorganize language ability, and these models prescribe different approaches for the use of TMS in persons with aphasia. The first and least controversial of these posits that after focal injury of the language dominant hemisphere in stroke, perilesional regions of the left hemisphere (i.e., regions near the area damaged by stroke) are recruited to subserve reorganized language function. This model suggests that one promising approach using TMS would be to excite brain activity in these perilesional left hemisphere regions, an approach successfully taken by Dammekens and colleagues when they applied high-frequency (10 Hz) TMS to the left inferior frontal gyrus a chronic aphasic patient that sustained a stroke affecting the left hemisphere. They found that patient's performance dramatically improved in repetition and naming tasks. These improvements lasted at least 4 months after stimulation (Dammekens et al., 2014).

Other models of stroke recovery focus on the observation that the right hemisphere increases its activation during language tasks in the setting of left-hemisphere strokes. However, the specific role played by the right hemisphere remains controversial. One often-invoked account suggests that right hemisphere activation in the setting of left hemisphere stroke and aphasia is deleterious to language performance. Following severe damage to the left hemisphere, there is thought to be a reduction of transcallosal inhibition, which leads to activation of the right hemisphere. The remodeled language network that arises from the recruitment of the right hemisphere is thought by some to result in inefficient functioning when compared to the activity of the left hemisphere prior to injury (Turkeltaub et al., 2011a). The deleterious nature of the right hemisphere is thought to be due to interhemisphereic inhibition preventing perilesional left hemisphere areas from resuming their role in language production (Belin et al., 1996; Rosen et al., 2000; Shimizu et al., 2002; Martin et al., 2004). This model suggests that inhibiting right hemisphere structures would be an appropriate strategy for enhancing language recovery.

A study by Naeser and colleagues targeted a focal segment of the non-damaged right hemisphere and applied low-frequency rTMS. They found that suppressing the right pars triangularis (PTr) with low-frequency (1 Hz) rTMS led to a significant increase in picture naming accuracy and a decrease in response time. Suppressing the right pars opercularis (Pop), on the other hand, led to a significant increase in response time and no change in the number of pictures named accurately (Naeser et al., 2011). This suggests certain specific areas of the right hemisphere may need to be inhibited for optimal language recovery. Further supporting the utility of inhibiting the right hemisphere, (Khedr et al., 2014) employed a bi-hemispheric stimulation paradigm. In their study Broca's area was stimulated with high-frequency rTMS (20 Hz) and the right hemisphere homolog of Broca's area was inhibited with low-frequency rTMS (1 Hz). In this sham controlled study, patients demonstrated improvement in language that was sustained at the 2-month follow up session (Khedr et al., 2014).

A third model is that the right hemisphere's main role is compensatory in nature. Because TMS studies have largely focused on the interhemispheric inhibition model, there is little data from magnetic brain stimulation studies to support this, however, it is supported by a variety of other accounts including case studies and functional neuroimaging reports (Turkeltaub et al., 2012).

Evidence, however, also supports the notion that these models are not mutually exclusive. Taking into consideration the evidence supporting left and right hemisphere models of aphasia recovery, it is likely that the process is dynamic and involves neuroplastic changes in both hemispheres. Utilizing studies of activation patterns and lesion distribution data, Heiss and Thiel presented a hierarchical model that explored three primary patterns of lesion distribution facilitating recovery in post-stroke aphasia. The first model proposes the restoration of the original activation pattern in the dominant hemisphere. In order to activate perilesional areas, the second model proposes the inhibition of recruited areas in the contralesional hemisphere. The last model, presents the idea of interhemispheric compensation, wherein the recruited homotopic areas are beneficial to language recovery (Heiss and Thiel, 2006).

A meta-analysis of functional imaging literature in patients with aphasia conducted by our lab utilized an activation likelihood estimation (ALE) on functional magnetic resonance imaging (fMRI) data to determine areas of language task-related activity in chronic non-fluent aphasic patients. This study demonstrated a bilateral network of activation in the aphasic population. We also compared patterns of functional activity between sites in patients with chronic aphasia and healthy individuals, in order to make inferences about the roles that activated regions may play in patients with aphasia. Areas recruited in the right hemisphere by aphasic patients by and large mirrored those activated in the left hemisphere of the healthy controls in both location and activation pattern, with the exception of one area, the right pars triangularis (PTr). The right PTr exhibited a different pattern of functional activity than the left hemisphere, suggesting some degree of discordance with the function of left hemisphere language areas in healthy subjects (Turkeltaub et al., 2011a). While this finding in the right PTr suggests that there are areas that do not contribute efficiently to language performance, the finding that most other active areas in the right hemisphere mirrored those of the left suggested that the activity of the right hemisphere is not entirely detrimental to the language recovery process, and may be largely compensatory.

Further exploring the role of the right hemisphere, our lab published a case report of a patient with sequential left and right hemisphere strokes. Following the initial stroke affecting the left hemisphere, the subject enrolled in a TMS trial where she received 10 daily sessions of inhibitory TMS to the right PTr. Inhibitory TMS to the right PTr led to improvements in naming that were maintained 2 months after stimulation. At that time, fMRI confirmed a reduction in the activity of the right PTr. Three months after TMS the patient sustained a second ischemic stroke affecting the right hemisphere, which resulted in worsening aphasia in the absence of other clinical deficits, such as weakness or sensory loss. Behavioral tests confirmed that language function was affected more so than other cognitive domains (Turkeltaub et al., 2011b). The improvements in language function seen after inhibitory TMS to the right PTr and the subsequent worsening after a stroke affecting the right hemisphere supports the idea that the role of the right hemisphere in aphasia recovery is not monolithic, and that some areas of the right hemisphere are beneficial to the recovery of language in post-stroke aphasia.

Language reorganization is a dynamic process and different mechanisms of recovery may be important to different degrees at different times in the post-stroke population. TMS studies, in general, have ranged in their chronicity from subacute to chronic. For example, the studies by Ren et al. and Dammekens et al. engage subjects ranging from 9.4 days to 75 months. A study by Saur and colleagues demonstrated that, after stroke, aphasic patients exhibit increased brain activation on the right during the subacute phase of recovery, followed by a leftward shift in the chronic phase (Saur et al., 2006). These dynamic shifts in language-associated brain activity could theoretically inform which brain stimulation approach is likely to be the most useful in different populations at different times. However, to date no investigation has directly investigated the efficacy of different sides of stimulation at different phases of post-stroke recovery. A recent meta-analysis by Shah-Basak et al. (2016) investigated the efficacy of rTMS and tDCS, including a subanalysis that explored stimulation at different post-stroke phases. They found comparable effect sizes in subacute and chronic studies, though the majority of studies employed a similar approach: low frequency stimulation of the right hemisphere. Whether patients in the subacute or acute phases of stroke recovery would benefit optimally from a different rTMS approach remains an open question.

### tDCS

Evidence now suggests that tDCS, may also improve aspects of language production in persons with chronic left hemisphere stroke and non-fluent aphasia (Monti et al., 2008; Baker et al., 2010; Fiori et al., 2011; Fridriksson et al., 2011; Medina et al., 2012). Utilizing a multimodal approach, studies have explored whether different stimulation approaches might be more beneficial for different patients (Datta et al., 2011; Shah-Basak et al., 2015). For example, a study conducted by our lab investigated whether individualized tDCS with different montages could result in lasting language recovery. In this two-phase study chronic patients were first stimulated with four different montages, which included placement of the anode and the cathode over the right and left frontal lobes on different days. Then in the second phase, subjects were randomized into a sham or treatment arm. In the treatment arm patients were stimulated with the montage identified as being most effective in Phase 1 of the study for 10 consecutive days. If randomized into the sham arm subjects received sham stimulation for 10 consecutive days and then crossed over into the treatment arm. Language ability significantly improved at the 2-week and 2-month follow-up time point. Seven out of 12 subjects responded well to a least 1 montage, but the optimal electrode montage across subjects was inconsistent, suggesting not only that tDCS can effectively modulate language recovery, but also that the optimal current pattern for facilitating lasting recovery may differ between patients (Shah-Basak et al., 2015).

In support of the notion that language recovery can be driven by the activation of perilesional brain areas, several studies have demonstrated that activating the left hemisphere with excitatory anodal tDCS has been associated with lasting improvements in language (Flöel et al., 2008; Baker et al., 2010; Fiori et al., 2011; Marangolo et al., 2013; Santos et al., 2013; Meinzer et al., 2014; Vestito et al., 2014; Wu et al., 2015). Baker and colleagues evaluated the effect of tDCS on chronic aphasic patients. Subjects received 5 days of anodal tDCS and 5 days of sham stimulation to the left frontal cortex. During stimulation patients performed a computerized anomia treatment. Significant improvement was observed in naming accuracy after anodal tDCS compared to sham (Baker et al., 2010). A more recent study by Wu and colleagues demonstrated that following anodal stimulation to the left hemisphere, chronic and sub-acute patients demonstrated marked improvements in picture naming and auditory comprehension compared to sham stimulation (Wu et al., 2015).

Regarding the second model, in which the right hemisphere is thought to be deleterious to the language recovery process, some groups have explored inhibitory paradigms. Cathodal tDCS (ctDCS), for example, is thought to decrease excitability of stimulated cortical sites (Nitsche and Paulus, 2001). Kang and colleagues designed a study to evaluate the effect of ctDCS over the right hemisphere in the homologous region to Broca's area in 10 patients with aphasia after stoke. They found that patients demonstrated a significant improvement in picture naming after the last tDCS session, as compared to no change after sham tDCS (Kang et al., 2011). In a different study with patients receiving ctDCS to the undamaged right hemisphere, Rosso and colleagues found improvements in picture naming were predicated on whether or not the arcuate fasciculus had been damaged. The authors also found that patients who had an abnormal interhemispheric balance responded well to inhibition of the undamaged hemisphere (Rosso et al., 2014), which suggests that the right hemisphere contribution to language recovery may, in some circumstances, be deleterious.

Integrating the first and second model, there is growing body of evidence supporting bi-hemispheric stimulation, wherein patients receive anodal tDCS to the left hemisphere and cathodal tDCS to the right hemisphere (Lee et al., 2013; Marangolo et al., 2014; Meinzer et al., 2014; Costa et al., 2015). Meinzer and colleagues conducted a study that contrasted the effect of anodal tDCS, dual hemisphere tDCS, and sham. They found that in both stimulation conditions word retrieval greatly improved (Meinzer et al., 2014). Other studies employing a bilateral stimulation approach support Meinzer, demonstrating that ctDCS to the right hemisphere Brodmann area 44/45 and anodal stimulation to left Brodmann 44/45 results in improved language performance (Costa et al., 2015).

Currently there are not many tDCS studies that directly support the third model of language plasticity, in which the contribution of the recruited right hemisphere is presumed to be beneficial to language recovery. However, one study by Vines and colleagues reported excitatory tDCS to the right inferior frontal gyrus resulted in improved fluency of speech (Vines et al., 2011).

Insofar as different mechanisms of plasticity may emerge in post-stroke aphasia at different times, there is reason to examine whether different tDCS approaches are more or less effective at different stages of recovery. Shah-Basak et al. (2016) examined the relevance of stroke chronicity in a meta-analysis, demonstrating that tDCS delivered in the subacute period resulted in a smaller and less consistent language benefit than stimulation applied in the chronic phase. However, it is unclear from this analysis whether the difference in effect was related to consistent differences in study design between subacute (all between-subject) and chronic (within-subject) investigations.

## TMS and tDCS in the treatment of PPA

### TMS

In primary progressive aphasia, the mechanisms by which language ability is represented in the brain are presumed to be different than those posited in post-stroke aphasia, insofar as these patients have cortical areas that are undergoing progressive atrophy, but are still believed to be involved in aspects of language function. Though these atrophic areas maintain some degree of activity in language tasks, over time they lose their processing efficiency and ultimately their functional and structural integrity (Ash et al., 2009; Grossman, 2010; Wilson et al., 2010; Rogalski et al., 2011)

Functional MRI performed during language tasks has been used to contrast the location and degree of brain activity of healthy subjects with that of subjects who have the non-fluent agrammatic variant of primary progressive aphasia (naPPA). Healthy controls were shown to activate both ventral regions of the left frontal lobe (which is associated with grammatical processing) and dorsal left frontal regions (which are associated with working memory). Patients with naPPA, however, only activated the dorsal portions of the left frontal lobe (Wilson et al., 2010). The absence of activity in the cortical regions associated with grammatical processing in the imaging data not only supports the clinical features of the disease, but also suggests that patients with naPPA utilize their remaining left hemisphere to engage in language production without significant right hemisphere recruitment. Of note, one study has previously suggested that a shift in temporal lobe activation from dominant to non-dominant hemisphere can be seen in PPA patients performing comprehension tasks (Vandenbulcke et al., 2005). The shift observed in this study was only observed in patients with a comorbid comprehension deficit (Vandenbulcke et al., 2005), which is not characteristic of non-fluent PPA. At this time the majority on (NIBS) studies in the PPA population focus on patients with fluency deficits. The overall interpretation and significance of this laterality shift remains unclear, particularly since it appears to correlate with worsening performance (Marsh and Hillis, 2006).

Currently, the conceptual approach taken by investigators studying PPA is not predicated on the idea that patients with different PPA subtypes are remapping language functions to novel networks, as seen in stroke patients, but rather that they are still using pre-existing neural areas, but with progressively declining efficiency. This suggests that a therapeutic strategy of facilitating brain activity in these pathologically weakened networks could be beneficial.

While low-frequency TMS is frequently implemented in studies with post-stroke aphasia, high-frequency excitatory rTMS (hf-rTMS) has more often been used in studies of PPA, based on the notion that the left hemisphere language network is broadly downregulated. Hf-rTMS (>5 Hz) has been shown to have an excitatory effect on cortical activity (Maeda et al., 2000). Studies have shown that left hemisphere hf-rTMS can augment a variety of language related tasks, including but not limited to picture naming (Töpper et al., 1998; Mottaghy et al., 1999) and oral word associations (Bridgers and Delaney, 1989). A case report by Finocchiaro and colleagues evaluated the effect of hf-rTMS to the left prefrontal cortex on language and memory. The study demonstrated that following hf-rTMS, subjects sustained a significant and lasting improvement in verb production (Finocchiaro et al., 2006). The study suggests that hf-rTMS to the dominant hemisphere directly strengthens processing within atrophic areas of the language network.

In a more recent study, Trebbastoni and colleagues demonstrated that when hf-TMS was applied to Broca's area and to underlying white matter bundles in the left dorsolateral prefrontal cortex (DLPFC), patients with logopenic variant PPA demonstrated improved accuracy of sentence production only after real stimulation (Trebbastoni et al., 2013). These results support the use of excitatory brain stimulation to enhance the remaining language function in atrophic cortical areas. Cotelli and colleagues conducted a similar study with progressive non-fluent aphasic patients. In their study, patients demonstrated improvement in action naming following hf-rTMS to the left and right DLPFC (Cotelli et al., 2012).

### tDCS

Similar to TMS in PPA, research concerning tDCS in PPA is currently sparse. The few studies that have explored the effects of tDCS in PPA have focused on the use of anodal (facilitative) stimulation of the left hemisphere, in order to enhance the activity of still existent but atrophied elements of the perisylvian language network.

Wang and colleagues reported a case in which anodal tDCS of the left posterior perisylvian region was applied in the morning and anodal stimulation of Broca's area was applied in the afternoon in a patient with non-fluent variant PPA. The paradigm resulted in improved auditory word comprehension, picture naming, oral word reading, and word repetition (Wang et al., 2013). More recently, Tsapkini and colleagues explored the effects of anodal tDCS of the left inferior frontal gyrus paired with a behavioral spelling intervention in patients with primary progressive aphasia. Using a within-subject crossover design, they were able to compare a tDCS + spelling intervention paradigm to a sham + spelling intervention paradigm. They found that spelling ability in patients in both the sham group and the stimulation group improved, however, patients that received tDCS + spelling intervention had a persistent improvement at least 2 months after stimulation (Tsapkini et al., 2014). All studies to date have focused on increasing activity the dominant hemisphere. Consistent with differences in models of language representation between post-stroke aphasia and PPAs, no investigators to date have attempted to enhance language abilities in patients with PPA by applying excitatory stimulation to the non-dominant hemisphere.

## Discussion

**Figure 1:**
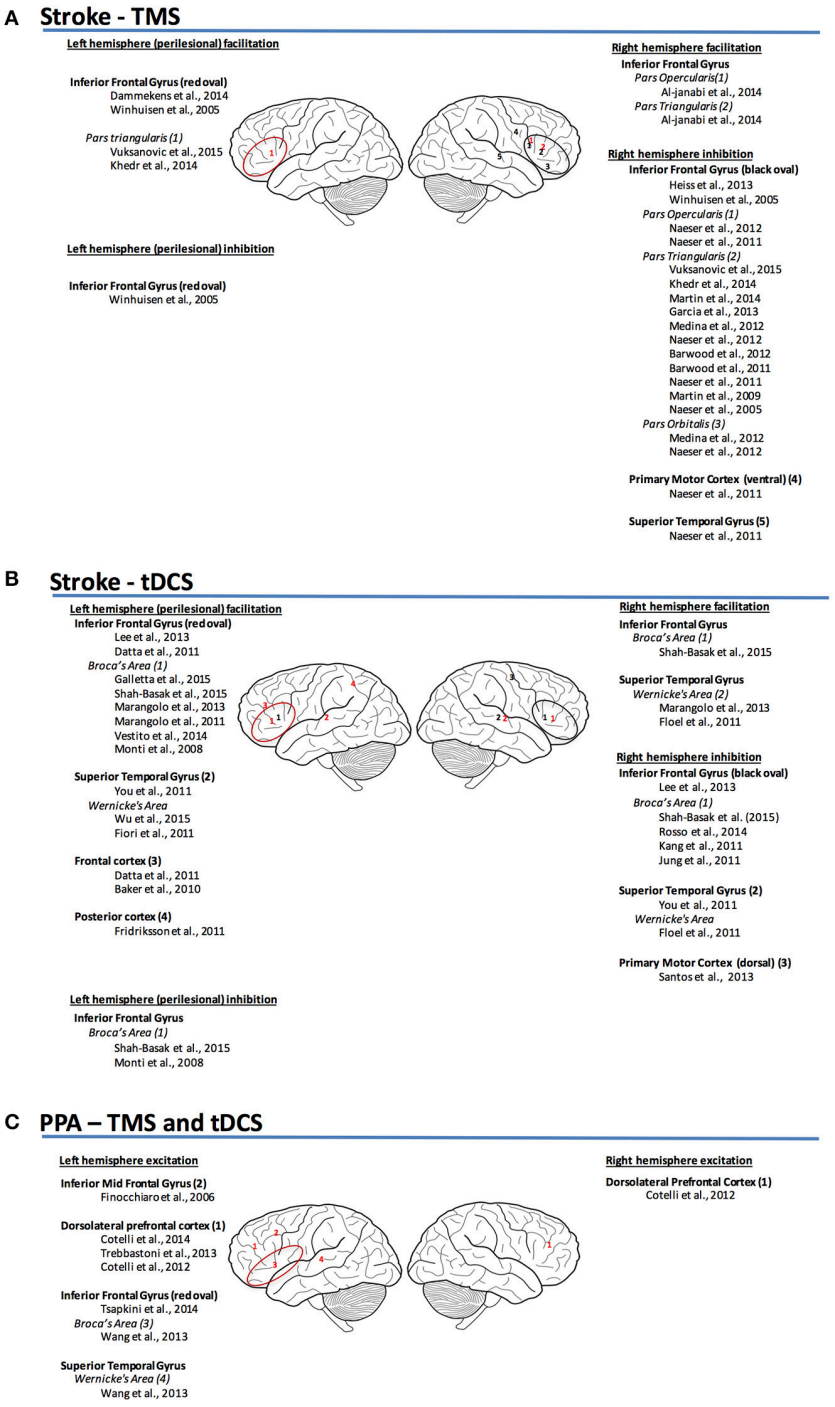
**(A)** Sites and mechanism of stimulation for TMS post-stroke aphasia (Garcia et al., 2013; Vuksanović et al., 2015). **(B)** Sites and mechanism of stimulation for tDCS post-stroke aphasia. **(C)** Sites and mechanism of stimulation for TMS and tDCS for PPA (red, facilitation; black, inhibition).

The differences in the mechanisms of language representation between post-stroke aphasia and PPA (Figure 1) inform us with respect to the factors and properties that may define and limit the success of different neuromodulation therapies. Additionally, these differences may also point to likely future directions for research in neuromodulation therapies for these two etiologies of aphasia.

The theorized mechanisms for recovery in post-stroke aphasia are largely predicated on the notion that regions of the brain that had previously been uninvolved in language processing or involved in a different way may take on new roles that had previously been subserved by the dominant hemisphere's perisylvian structures (Belin et al., 1996; Rosen et al., 2000; Shimizu et al., 2002; Martin et al., 2004; Heiss and Thiel, 2006; Turkeltaub et al., 2011a; Anglade et al., 2014). Intuitively, the effectiveness of this mechanism is constrained by at least two basic properties. The first is the extent to which the regions of brain that are being recruited to compensate for lost functions harbor the same kinds of computational affordances and capacities that injured regions had possessed. In other words, how well are the brain areas that are reassigned to take over for lost areas able to perform the same tasks as the areas that were lost to injury? The second property is the degree of overlap in anatomic or functional connectivity between recruited compensatory brain regions and areas that have been injured. In other words, in order to do the job of a lesioned area of brain, it is beneficial to have existing connections to the areas that the old region used to connect to. With respect to both of these properties, perilesional and right hemisphere homotopes of the injured left hemisphere areas are reasonable targets. However, these may not be the only good anatomic candidates for neuroplastic change in patients with aphasia due to stroke. For example, a recent study by (Xing et al., 2016) suggests that increased right temporoparietal gray matter volume is associated with improved language performance in patients with post-stroke aphasia even when those regions are not homotopic to the patients' lesions (Xing et al., 2016).

Other studies favor the recovery of the residual left hemisphere, suggesting that the contributions of the right hemisphere are ineffective (Belin et al., 1996; Rosen et al., 2000; Shimizu et al., 2002; Martin et al., 2004). One property thought to influence the efficacy of left hemisphere perilesional compensation relative to right hemisphere recruitment with respect to language recovery is lesion size (Heiss and Thiel, 2006). It has been suggested that smaller lesions of the left hemisphere are more associated with left hemisphere/perilesional recruitment and large left hemisphere lesions are associated with more right hemisphere activity (Heiss and Thiel, 2006), although some recent lesion evidence may weigh against this view (Turkeltaub, 2015). Another property that has been shown to influence the compensatory potential of the right hemisphere worth considering is lesion location (Anglade et al., 2014). A recent review by Saur and colleagues explored the possibility that the involvement of right hemisphere homologs of left hemisphere language regions may depend on the degree of premorbid lateralization. That is, patients with more bilaterally distributed language representation prior to their stroke may be better able to recruit the right hemisphere for additional language processing in the setting of left hemisphere injury (Saur and Hartwigsen, 2012).

If we accept that these properties probably influence the compensatory potential of undamaged brain regions after stroke, it seems likely that the success of neuromodulation interventions in post-stroke aphasia rests on finding the best candidate targets to stimulate (i.e., those that have the necessary computational affordances and connectivity). Existing TMS studies have already provided some evidence of this. Our data and that of other investigators demonstrate that the site of stimulation in the brain is pivotal in post-stroke aphasia (Hamilton et al., 2010; Ren et al., 2014).

Moreover, as tools for characterizing connectivity in the brain become more sophisticated, models of language reorganization are being informed by an increasingly nuanced understanding of brain networks, which may in turn inform therapeutic neuromodulation approaches. Post-stroke aphasia studies have demonstrated that an increase in frontoparietal integration was correlated with language recovery (Sharp et al., 2010). Naming treatment studies have also demonstrated alterations in functional connectivity specific to a shift from activity in the right middle temporal gyrus to the left middle temporal and supramagrinal gyrus after treatment (van Hees et al., 2014). A recent study by Yang and colleagues demonstrated that patients with aphasia exhibited increased regional activity in the contralesional mesial temporal and lateral temporal cortices compared to aged matched controls (Yang et al., 2016). Taken together the post-stroke studies suggest that increased integration and/or activity in frontoparietal or contralesional temporal regions is associated with language recovery. To date, there are fewer functional connectivity studies in the PPA population compared to post-stroke aphasia. A recent study by Mandelli and colleagues evaluated functional connectivity in healthy controls and patients with PPA. Unlike the observed increase in contralateral activity seen in the post-stroke population, the authors demonstrated marked areas of reduced strength of functional connectivity to the dorsal portion of the opercular region of the left inferior frontal gyrus (Mandelli et al., 2016). Overall the existing functional connectivity literature for the two types of non-fluent aphasia suggests that that language-related changes in connectivity in PPA are relatively isolated, while post-stroke shifts in activity are more bilaterally distributed. This broader distribution supports consideration of multiple mechanisms of recovery and multiple montages as targets for neuromodulation.

We already have tools such as BOLD fMRI that are capable of suggesting which areas of the brain are capable of taking on compensatory roles with respect to language processing in patient with aphasia (Carter et al., 2012; Havsteen et al., 2013). Excitingly, more recent tools like advanced structural imaging, diffusion tensor imaging (DTI), diffusion spectrum imaging (DSI) and computational network modeling can be used to tell us which brain regions are the best candidates for compensation by characterizing their connectivity and network properties, respectively (Dijkhuizen et al., 2012; Ovadia-Caro et al., 2013). Moreover, a recent study by Cipollari and colleagues demonstrated that, in the post-stroke aphasic population, tDCS can be paired with TMS-EEG to target and modulate specific areas of excitability, resulting in specific language improvements (Cipollari et al., 2015). Armed with this knowledge, it may eventually be possible to use focused neuromodulation techniques like TMS and tDCS to entrain optimal regions into networks for language recovery (regions that may be potentially better suited for language processing than those that the brain may have otherwise recruited in the absence of neuromodulation).

The mechanisms of language representation and loss in PPAs present with different kinds of limitations and opportunities. PPAs are characterized by the slow, distributed degeneration of cellular units within the language system. This process may initially be characterized by a *graceful degradation*, wherein the language system and other affected systems are initially robust against the behavioral effects of diffuse ongoing injury. This is borne out by evidence from neuropathology studies, which demonstrate that in neurodegenerative dementias like AD and FTD, neural injury occurs for years and perhaps decades before patients become symptomatic (Grossman, 2010). Eventually, however, language systems and other behaviorally relevant networks cannot tolerate the distributed network failure created by neurodegenerative dementias.

Evidence indicates that neuromodulation techniques can be used to enhance neuronal plasticity at the level of synaptic communication (Bindman et al., 1964; Bliss and Lomo, 1973; Hattori et al., 1990; Moriwaki, 1991; Liebetanz et al., 2002). The onset of aphasia is less clearly defined in PPA as compared to stroke, so it is challenging to define a precise timeframe for intervention. In order to influence neuronal plasticity in the residually functioning networks, it is reasonable that the ideal timeframe for stimulation would be close to symptom recognition and diagnosis. We can conceptualize the effect of brain stimulation in PPA as a race to create neuroplastic changes that improve the strength of connections within language networks. These intervention-induced changes may work to partially counter the behavioral effects of distributed degradation of the language network at the cell and circuit level, in effect permitting the language network to do more with less. Therefore, it stands to reason that the efficacy of neuromodulation approaches in PPA would depend, at least to some extent, on the effectiveness of the intervention in inducing distributed increases in compensatory plasticity.

In light of this, our prediction is that, unlike stroke, the near future of neuromodulation in PPA is not going to be focused on identifying the best sites to stimulate in conjunction with behavioral therapies, but rather on determining the parameters and paired behavioral therapies that are best suited for inducing plastic changes that reinforce the robustness of already-connected but weakening language systems. While this may be attainable with TMS, tDCS seems well suited for these demands, owing to its comparatively low spatial resolution. Additionally, tDCS is a technique of interest because it has been shown that pairing stimulation with behavioral training can create persistent changes in neural activity and behavior that are specific to the kinds of activities that were employed during stimulation (Gill et al., 2015). Other related neuromodulation technologies like transcranial alternating current stimulation (tACS) and transcranial random noise stimulation (tRNS) may also have promising effects on network plasticity (Fertonani et al., 2011; Antal and Paulus, 2013) that may prove beneficial in these patient populations.

Given the presumed mechanism of language representation in PPA discussed above, it is not clear how inhibitory stimulation would be useful in PPAs. Unlike stroke, there is currently no theoretical role for suppressing components of the language network based on current evidence. Rather, the evidence favors using excitatory stimulation to create a more plastic and robust system. Again, like stroke, the development of imaging tools like resting state functional connectivity MRI (rs-fcMRI) allow us to discern change in the robustness of networks which will prove valuable for helping us understand the effects and efficacy of neuromodulation technologies in PPA.

## Conclusion

Given the burden of suffering imposed on patients by both post-stroke aphasia and PPAs, and the promising results seen in neuromodulation studies of aphasia to date, larger and more extensive future clinical studies involving TMS, tDCS, and related NIBS approaches as treatments for both etiologies aphasia seem likely. These future investigations will need to take into account how pathologic processes could be strong determinants of brain stimulation effects, and could predict the optimal technology (TMS, tDCS, or other), target sites, and stimulation parameters for neuromodulation therapies. Importantly, the notion that the neural mechanisms of injury and neuroplastic reorganization should influence decision-making around the use of NIBS is not specific to the aphasia treatment. Models of normal and abnormal brain function are becoming increasingly complex, accurate, and predictive owing to continuing advances in cognitive neuroscience, neuroimaging, and network science. It is clear that clinicians and researchers hoping to pursue investigations involving the use of TMS, tDCS, or other non-invasive neuromodulation technologies to remediate cognitive deficits beyond aphasia as well as other symptoms associated with neurologic disease will need to devise approaches that align well with existing and future models of brain injury, plasticity, and recovery.

## Author contributions

CN contributed substantially to the conception of the work, was responsible for drafting the manuscript, and agrees to be accountable for all aspects of the work in ensuring that questions related to the accuracy or integrity of any part of the work are appropriately investigated and resolved. RH contributed substantially to the conception of the work, was responsible for revising the manuscript critically for important intellectual content, and agrees to be accountable for all aspects of the work in ensuring that questions related to the accuracy or integrity of any part of the work are appropriately investigated and resolved.

## Funding

Through the Master in Translational Research program CN received the CTSA TL1 award (TL1TR000138).

### Conflict of interest statement

The authors declare that the research was conducted in the absence of any commercial or financial relationships that could be construed as a potential conflict of interest.

## References

[B1] Abel S. Weiller C. Huber W. Willmes K. Specht K. (2015). Therapy-induced brain reorganization patterns in aphasia. Brain 138, 1097–1112. 10.1093/brain/awv022 25688082

[B2] Abo M. Kakuda W. Watanabe M. Morooka A. Kawakami K. Senoo A. (2012). Effectiveness of low-frequency rTMS and intensive speech therapy in post-stroke patients with aphasia: a pilot study based on evaluation by fMRI in relation to type of aphasia. Eur. Neurol. 68, 199–208. 10.1159/000338773 22948550

[B3] Alexander M. P. (1997). Aphasia: clinical and anatomical aspects, in Behavioral Neurology and Neuropsychology, eds Feinberg T. E. Farah M. J. (New York, NY: McGraw-Hill), 133–49.

[B4] Al-Janabi S. Nickels L. A. Sowman P. F. Burianová H. Merrett D. L. Thompson W. F. (2014). Augmenting melodic intonation therapy with non-invasive brain stimulation to treat impaired left-hemisphere function: two case studies. Front. Psychol. 5:37. 10.3389/fpsyg.2014.00037 24550864 PMC3912988

[B5] Ambrus G. G. Al-Moyed H. Chaieb L. Sarp L. Antal A. Paulus W. (2012). The fade-in–short stimulation–fade out approach to sham tDCS–reliable at 1 mA for naïve and experienced subjects, but not investigators. Brain Stimul. 5, 499–504. 10.1016/j.brs.2011.12.001 22405745

[B6] American Heart Association (2016). Available online at: http://circ.ahajournals.org/content/133/4/e38

[B7] Anglade C. Thiel A. Ansaldo A. I. (2014). The complementary role of the cerebral hemispheres in recovery from aphasia after stroke: a critical review of literature. Brain Inj. 28, 138–145. 10.3109/02699052.2013.859734 24456053

[B8] Antal A. Paulus W. (2013). Transcranial alternating current stimulation (tACS). Front. Hum. Neurosci. 7:317. 10.3389/fnhum.2013.00317 23825454 PMC3695369

[B9] Ash S. Moore P. Vesely L. Gunawardena D. McMillan C. Anderson C.. (2009). Non-fluent speech in frontotemporal lobar degeneration. J. Neurolinguist. 22, 370–383. 10.1016/j.jneuroling.2008.12.001 22180700 PMC3238501

[B10] Baker J. M. Rorden C. Fridriksson J. (2010). Using transcranial direct-current stimulation to treat stroke patients with aphasia. Stroke 41, 1229–1236. 10.1161/STROKEAHA.109.576785 20395612 PMC2876210

[B11] Barwood C. H. Murdoch B. E. Riek S. O'Sullivan J. D. Wong A. Lloyd D.. (2013). Long term language recovery subsequent to low frequency rTMS in chronic non-fluent aphasia. Neuro Rehabil. 32, 915–928. 10.3233/NRE-130915 23867417

[B12] Barwood C. H. Murdoch B. E. Whelan B. M. Lloyd D. Riek S. O' Sullivan J. D.. (2011). Improved language performance subsequent to low-frequency rTMS in patients with chronic non-fluent aphasia post-stroke. Eur. J. Neurol. 18, 935–943. 10.1111/j.1468-1331.2010.03284.x 21138505

[B13] Barwood C. H. Murdoch B. E. Whelan B. M. Lloyd D. Riek S. O'Sullivan J. D.. (2012). Improved receptive and expressive language abilities in nonfluent aphasic stroke patients after application of rTMS: an open protocol case series. Brain Stimul. 3, 274–286. 10.1016/j.brs.2011.03.005 22037124

[B14] Basso A. Marangolo P. (2000). Cognitive rehabilitation: the emperor's new clothes? Neuropsychol. Rehab. 10, 219–229. 10.1080/096020100389138

[B15] Belin P. Van Eeckhout P. Zilbovicious M. Remy P. François C. Guillaume S.. (1996). Recovery from nonfluent aphasia after melodic intonation therapy: a PET study. Neurology 47, 1504–1511. 10.1212/WNL.47.6.1504 8960735

[B16] Bernal B. Ardila A. (2009). The role of the arcuate fasciculus in conduction aphasia. Brain 132, 2309–2316. 10.1093/brain/awp206 19690094

[B17] Binder J. (2015). The Wernicke area: Modern evidence and a reinterpretation. Neurology 85, 2170–2175. 10.1212/WNL.0000000000002219 26567270 PMC4691684

[B18] Bindman L. J. Lippold O. C. Redfearn J. W. (1964). The action of brief polarizing currents on the cerebral cortex of the rat (1) during current flow and (2) in the production of long-lasting after-effects. J. Physiol. 172, 369–382. 10.1113/jphysiol.1964.sp007425 14199369 PMC1368854

[B19] Blesneag A. V. Slavoaca D. F. Popa L. Stan A. D. Jemna N. Isai Moldovan F.. (2015). Low-frequency rTMS in patients with subacute ischemic stroke: clinical evaluation of short and long-term outcomes and neurophysiological assessment of cortical excitability. J. Med. Life 8, 378–387. 26351545 PMC4556924

[B20] Bliss T. V. Lomo T. (1973). Long-lasting potentiation of synaptic transmission in the dentate area of the anaesthetized rabbit following stimulation of the perforant path. J. Physiol. (Lond). 232, 331–356. 10.1113/jphysiol.1973.sp010273 4727084 PMC1350458

[B21] Boggio P. S. Rigonatti S. P. Ribeiro R. B. Myczkowski M. L. Nitsche M. A. Pascual-Leone A.. (2008). A randomized, double-blind clinical trial on the efficacy of cortical direct current stimulation for the treatment of major depression. Int. J. Neuropsychopharmacol. 11, 249–254. 10.1017/S1461145707007833 17559710 PMC3372849

[B22] Bolognini N. Pascual-Leone A. Fregni F. (2009). Using non-invasive brain stimulation to augment motor training-induced plasticity. J. Neuroeng. Rehabil. 6:8. 10.1186/1743-0003-6-8 19292910 PMC2667408

[B23] Bridgers S. L. Delaney R. C. (1989). Transcranial magnetic stimulation: an assessment of cognitive and other cerebral effects. Neurology 39:417. 10.1212/WNL.39.3.417 2927652

[B24] Broca P. (1861). Perte de la parole, ramollissement chronique et destruction partielle du lobe antérieure gauche du cerveau. Bull Soc. Anthrop. Paris 2, 235–238.

[B25] Brunoni A. R. Ferrucci R. Bortolomasi M. Vergari M. Tadini L. Boggio P. S.. (2011). Transcranial direct current stimulation (tDCS) in unipolar vs. bipolar depressive disorder. Prog. Neuropsychopharmacol. Biol. Psychiatry 35, 96–101. 10.1016/j.pnpbp.2010.09.010 20854868

[B26] Brunoni A. R. Ferrucci R. Fregni F. Boggio P. S. Priori A. (2012). Transcranial direct current stimulation for the treatment of major depressive disorder: a summary of preclinical, clinical and translational findings. Prog. Neuropsychopharmacol. Biol. Psychiatry 39, 9–16. 10.1016/j.pnpbp.2012.05.016 22651961

[B27] Carter A. R. Shulman G. L. Corbetta M. (2012). Why use a connectivity-based approach to study stroke and recovery of function? Neuroimage 62, 2271–2280. 10.1016/j.neuroimage.2012.02.070 22414990 PMC3733251

[B28] Charles D. Olm C. Powers J. Ash S. Irwin D. J. McMillan C. T.. (2014). Grammatical comprehension deficits in non-fluent/agrammatic primary progressive aphasia. J. Neurol. Neurosurg. Psychiatry 85, 249–256. 10.1136/jnnp-2013-305749 24039027 PMC3925677

[B29] Cipollari S. Veniero D. Razzano C. Caltagirone C. Koch G. Marangolo P. (2015). Combining TMS-EEG with transcranial direct current stimulation language treatment in aphasia. Expert Rev. Neurother. 15, 833–845. 10.1586/14737175.2015.1049998 26109229

[B30] Costa V. Giglia G. Brighina F. Indovino S. Fierro B. (2015). Ipsilesional and contralesional regions participate in the improvement of post-stroke aphasia: a transcranial direct current stimulation study. Neurocase 21, 479–488. 10.1080/13554794.2014.927508 24957199

[B31] Cotelli M. Manenti R. Alberici A. Brambilla M. Cosseddu M. Zanetti O.. (2012). Prefrontal cortex rTMS enhances action naming in progressive non-fluent aphasia. Eur. J. Neurol. 19, 1404–1412. 10.1111/j.1468-1331.2012.03699.x 22435956

[B32] Cotelli M. Manenti R. Petesi M. Brambilla M. Cosseddu M. Zanetti O.. (2014). Treatment of primary progressive aphasias by transcranial direct current stimulation combined with language training. J. Alzheimers. Dis. 39, 799–808. 10.3233/JAD-131427 24296814

[B33] Dammekens E. Vanneste S. Ost J. De Ridder D. (2014). Neural correlates of high frequency repetitive transcranial magnetic stimulation improvement in post-stroke non-fluent aphasia: a case study. Neurocase 20, 1–9. 10.1080/13554794.2012.713493 22963195

[B34] Datta A. Baker J. M. Bikson M. Fridriksson J. (2011). Individualized model predicts brain current flow during transcranial direct-current stimulation treatment in responsive stroke patient. Brain Stimul. 4, 169–174. 10.1016/j.brs.2010.11.001 21777878 PMC3142347

[B35] Datta A. Bansal V. Diaz J. Patel J. Reato D. Bikson M. (2009). Gyri-precise head model of transcranial direct current stimulation: improved spatial focality using a ring electrode versus conventional rectangular pad. Brain Stimul. 2, 201–207. 10.1016/j.brs.2009.03.005 20648973 PMC2790295

[B36] Demirtas-Tatlidede A. Vahabzadeh-Hagh A. M. Bernabeu M. Tormos J. M. Pascual-Leone A. (2012). Noninvasive brain stimulation in traumatic brain injury. J. Head Trauma Rehabil. 27, 274–292. 10.1097/HTR.0b013e318217df55 21691215 PMC3342413

[B37] Dijkhuizen R. M. van der Marel K. Otte W. M. Hoff E. I. van der Zijden J. P. van der Toorn A.. (2012). Functional MRI and diffusion tensor imaging of brain reorganization after experimental stroke. Transl. Stroke Res. 3, 36–43. 10.1007/s12975-011-0143-8 22408692 PMC3284658

[B38] Elder G. J. Taylor J. P. (2014). Transcranial magnetic stimulation and transcranial direct current stimulation: treatments for cognitive and neuropsychiatric symptoms in the neurodegenerative dementias? Alzheimers. Res. Ther. 6:74. 10.1186/s13195-014-0074-1 25478032 PMC4255638

[B39] Fertonani A. Pirulli C. Miniussi C. (2011). Random noise stimulation improves neuroplasticity in perceptual learning. J. Neurosci. 31, 15416–15423. 10.1523/JNEUROSCI.2002-11.2011 22031888 PMC6703532

[B40] Finocchiaro C. Maimone M. Brighina F. Piccoli T. Giglia G. Fierro B. (2006). A case study of primary progressive aphasia: improvement on verbs after rTMS treatment. Neurocase 12, 317–321. 10.1080/13554790601126203 17182394

[B41] Fiori V. Coccia M. Marinelli C. V. Vecchi V. Bonifazi S. Ceravolo M. G.. (2011). Transcranial direct current stimulation improves word retrieval in healthy and nonfluent aphasic subjects. J. Cogn. Neurosci. 23, 2309–2323. 10.1162/jocn.2010.21579 20946060

[B42] Flöel A. Meinzer M. Kirstein R. Nijhof S. Deppe M. Knecht S.. (2011). Short-term anomia training and electrical brain stimulation. Stroke 42, 2065–2067. 10.1161/STROKEAHA.110.609032 21636820

[B43] Flöel A. Rösser N. Michka O. Knecht S. Breitenstein C. (2008). Noninvasive brain stimulation improves language learning. J. Cogn. Neurosci. 20, 1415–1422. 10.1162/jocn.2008.20098 18303984

[B44] Freitas C. Mondragón-Llorca H. Pascual-Leone A. (2011). Noninvasive brain stimulation in Alzheimer's disease: systematic review and perspectives for the future. Exp. Gerontol. 46, 611–627. 10.1016/j.exger.2011.04.001 21511025 PMC3589803

[B45] Fridriksson J. (2010). Preservation and modulation of specific left hemisphere regions is vital for treated recovery from anomia in stroke. J. Neurosci. 30, 11558–11564. 10.1523/JNEUROSCI.2227-10.2010 20810877 PMC2938788

[B46] Fridriksson J. Guo D. Fillmore P. Holland A. Rorden C. (2013). Damage to the anterior arcuate fasciculus predicts non-fluent speech production in aphasia. Brain 136, 3451–3460. 10.1093/brain/awt267 24131592 PMC3808690

[B47] Fridriksson J. Richardson J. D. Baker J. M. Rorden C. (2011). Transcranial direct current stimulation improves naming reaction time in fluent aphasia: a double-blind, sham-controlled study. Stroke 42, 819–821. 10.1161/STROKEAHA.110.600288 21233468 PMC8210639

[B48] Galletta E. E. Vogel-Eyny A. (2015). Translational treatment of aphasia combining neuromodulation and behavioral intervention for lexical retrieval: implications from a single case study. Front. Hum. Neurosci. 9:447. 10.3389/fnhum.2015.00447 26347634 PMC4541259

[B49] Garcia G. Norise C. Faseyitan O. Naeser M. A. Hamilton R. H. (2013). Utilizing repetitive transcranial magnetic stimulation to improve language function in stroke patients with chronic non-fluent aphasia. J. Vis. Exp. 77:e50228. 10.3791/50228 23852365 PMC3731176

[B50] Geschwind N. (1972). Language and the brain. Sci. Am. 226, 76–83. 10.1038/scientificamerican0472-76 5014017

[B51] Gill J. Shah-Basak P. P. Hamilton R. (2015). It's the thought that counts: examining the task-dependent effects of transcranial direct current stimulation on executive function. Brain Stimul. 8, 253–259. 10.1016/j.brs.2014.10.018 25465291

[B52] Gorno-Tempini M. L. Hillis A. E. Weintraub S. Kertesz A. Mendez M. Cappa S. F.. (2011). Classification of primary progressive aphasia and its variants. Neurology 76, 1006–1014. 10.1212/WNL.0b013e31821103e6 21325651 PMC3059138

[B53] Grossman M. (2010). Primary progressive aphasia: clinicopathological correlations. Nat. Rev. Neurol. 6, 88–97. 10.1038/nrneurol.2009.216 20139998 PMC3637977

[B54] Hamilton R. H. Chrysikou E. G. Coslett B. (2011). Mechanisms of aphasia recovery after stroke and the role of noninvasive brain stimulation. Brain Lang. 118, 40–50. 10.1016/j.bandl.2011.02.005 21459427 PMC3109088

[B55] Hamilton R. H. Sanders L. Benson J. Faseyitan O. Norise C. Naeser M.. (2010). Stimulating conversation: enhancement of elicited propositional speech in a patient with chronic non-fluent aphasia following transcranial magnetic stimulation. Brain Lang. 113, 45–50. 10.1016/j.bandl.2010.01.001 20159655 PMC2909623

[B56] Hasselmo M. E. (1995). Neuromodulation and cortical function: modeling the physiological basis of behavior. Behav. Brain Res. 67, 1–27. 10.1016/0166-4328(94)00113-T 7748496

[B57] Hattori Y. Moriwaki A. Hori Y. (1990). Biphasic effects of polarizing current on adenosine-sensitive generation of cyclic AMP in rat cerebral cortex. Neurosci. Lett. 116, 320–324. 10.1016/0304-3940(90)90094-P 2173816

[B58] Hausmann A. Weis C. Marksteiner J. Hinterhuber H. Humpel C. (2000). Chronic repetitive transcranial magnetic stimulation enhances c-fos in the parietal cortex and hippocampus. Brain Res. Mol. Brain Res. 76, 355–362. 10.1016/S0169-328X(00)00024-3 10762712

[B59] Havsteen I. Madsen K. H. Christensen H. Christensen A. Siebner H. R. (2013). Diagnostic approach to functional recovery: functional magnetic resonance imaging after stroke. Front. Neurol. Neurosci. 32, 9–25. 10.1159/000346408 23859959

[B60] Heiss W. D. Hartmann A. Rubi-Fessen I. Anglade C. Kracht L. Kessler J.. (2013). Noninvasive brain stimulation for treatment of right- and left-handed post-stroke aphasics. Cerebrovasc. Dis. 36, 363–372. 10.1159/000355499 24217362

[B61] Heiss W. D. Thiel A. (2006). A proposed regional hierarchy in recovery of post-stroke aphasia. Brain Lang. 98, 118–123. 10.1016/j.bandl.2006.02.002 16564566

[B62] Hodges J. R. Patterson K. (2007). Semantic dementia: a unique clinicopathological syndrome. Lancet Neurol. 6, 1004–1014. 10.1016/S1474-4422(07)70266-1 17945154

[B63] Hoffman R. E. Cavus I. (2002). Slow transcranial magnetic stimulation, long-term depotentiation, and brain hyperexcitability disorders. Am. J. Psychiatry 159, 1093–1102. 10.1176/appi.ajp.159.7.1093 12091184

[B64] Horvath J. C. Carter O. Forte J. D. (2016). No significant effect of transcranial direct current stimulation (tDCS) found on simple motor reaction time comparing 15 different simulation protocols. Neuropsychologia 21, 544–552. 10.1016/j.neuropsychologia.2016.09.017 27664296

[B65] Horvath J. C. Forte J. D. Carter O. (2015). Quantitative review finds no evidence of cognitive effects in healthy populations from single-session transcranial direct current stimulation (tDCS). Brain Stimul. 8, 535–550. 10.1016/j.brs.2015.01.400 25701175

[B66] Huang Y. Z. Edwards M. J. Rounis E. Bhatia K. P. Rothwell J. C. (2005). Theta burst stimulation of the human motor cortex. Neuron 45, 201–206. 10.1016/j.neuron.2004.12.033 15664172

[B67] Josephs K. A. Whitwell J. L. Duffy J. R. Vanvoorst W. A. Strand E. A. Hu W. T.. (2008). Progressive aphasia secondary to Alzheimer disease vs FTLD pathology. Neurology 70, 25–34. 10.1212/01.wnl.0000287073.12737.35 18166704 PMC2749307

[B68] Jung I. Y. Lim J. Y. Kang E. K. Sohn H. M. Paik N. J. (2011). The factors associated with good responses to speech therapy combined with transcranial direct current stimulation in post-stroke aphasic patients. Ann. Rehabil. Med. 35, 460–469. 10.5535/arm.2011.35.4.460 22506160 PMC3309227

[B69] Kang E. K. Kim Y. K. Sohn H. M. Cohen L. G. Paik N. J. (2011). Improved picture naming in aphasia patients treated with cathodal tDCS to inhibit the right Broca's homologue area. Restor. Neurol. Neurosci. 29, 141–152. 10.3233/RNN-2011-0587 21586821 PMC4886370

[B70] Khedr E. M. Abo El-Fetoh N. Ali A. M. El-Hammady D. H. Khalifa H. Atta H.. (2014). Dual-hemisphere repetitive transcranial magnetic stimulation for rehabilitation of post-stroke aphasia: a randomized, double-blind clinical trial. Neurorehabil. Neural Repair 28, 740–750. 10.1177/1545968314521009 24503205

[B71] Kreisler A. Godefroy O. Delmaire C. Debachy B. Leclercq M. Pruvo J. P.. (2000). The anatomy of aphasia revisited. Neurology 54, 1117–1123. 10.1212/WNL.54.5.1117 10720284

[B72] Kyrozis A. Potagas C. Ghika A. Tsimpouris P. K. Virvidaki E. S. Vemmos K. N. (2009). Incidence and predictors of post-stroke aphasia: the Arcadia Stroke Registry. Eur. J. Neurol. 16, 733–739. 10.1111/j.1468-1331.2009.02580.x 19475755

[B73] Laska A. C. Hellblom A. Murray V. Kahan T. Von Arbin M. (2001). Aphasia in acute stroke and relation to outcome. J. Intern. Med. 249, 413–422. 10.1046/j.1365-2796.2001.00812.x 11350565

[B74] Lee S. Y. Cheon H. J. Yoon K. J. Chang W. H. Kim Y. H. (2013). Effects of dual transcranial direct current stimulation for aphasia in chronic stroke patients. Ann. Rehabil. Med. 37, 603–610. 10.5535/arm.2013.37.5.603 24233579 PMC3825935

[B75] Li Y. Qu Y. Yuan M. Du T. (2015). Low-frequency repetitive transcranial magnetic stimulation for patients with aphasia after stoke: a meta-analysis. J. Rehabil. Med. 47, 675–681. 10.2340/16501977-1988 26181486

[B76] Liebetanz D. Nitsche M. A. Tergau F. Paulus W. (2002). Pharmacological approach to the mechanisms of transcranial DC-stimulation-induced after-effects of human motor cortex excitability. Brain 125, 2238–2247. 10.1093/brain/awf238 12244081

[B77] Maeda F. Keenan J. P. Tormos J. M. Topka H. Pascual-Leone A. (2000). Interindividual variability of the modulatory effects of repetitive transcranial magnetic stimulation on cortical excitability. Exp. Brain Res. 133, 425–430. 10.1007/s002210000432 10985677

[B78] Mandelli M. L. Vilaplana E. Brown J. A. Hubbard H. I. Binney R. J. Attygalle S.. (2016). Healthy brain connectivity predicts atrophy progression in non-fluent variant of primary progressive aphasia. Brain 139, 2778–2791. 10.1093/brain/aww195 27497488 PMC5035819

[B79] Marangolo P. Fiori V. Calpagnano M. A. Campana S. Razzano C. Caltagirone C.. (2013). tDCS over the left inferior frontal cortex improves speech production in aphasia. Front. Hum. Neurosci. 7:539. 10.3389/fnhum.2013.00539 24046740 PMC3764371

[B80] Marangolo P. Fiori V. Gelfo F. Shofany J. Razzano C. Caltagirone C.. (2014). Bihemispheric tDCS enhances language recovery but does not alter BDNF levels in chronic aphasic patients. Restor. Neurol. Neurosci. 32, 367–379. 10.3233/RNN-130323 24398720

[B81] Marangolo P. Marinelli C. V. Bonifazi S. Fiori V. Ceravolo M. G. Provinciali L.. (2011). Electrical stimulation over the left inferior frontal gyrus (IFG) determines long-term effects in the recovery of speech apraxia in three chronic aphasics. Behav. Brain Res. 225, 498–504. 10.1016/j.bbr.2011.08.008 21856336

[B82] Marsh E. B. Hillis A. E. (2006). Recovery from aphasia following brain injury: the role of reorganization. Prog. Brain Res. 157, 143–156. 10.1016/S0079-6123(06)57009-8 17046670

[B83] Martin P. I. Naeser M. A. Ho M. Doron K. W. Kurland J. Kaplan J.. (2009). Overt naming fMRI pre- and post-TMS: two nonfluent aphasia patients, with and without improved naming post-TMS. Brain Lang. 111, 20–35. 10.1016/j.bandl.2009.07.007 19695692 PMC2803355

[B84] Martin P. I. Naeser M. A. Theoret H. Tormos J. M. Nicholas M. Kurland J.. (2004). Transcranial magnetic stimulation as a complementary treatment for aphasia. Semin. Speech Lang. 25, 181–191. 10.1055/s-2004-825654 15118944

[B85] Martin P. I. Treglia E. Naeser M. A. Ho M. D. Baker E. H. Martin E. G.. (2014). Language improvements after TMS plus modified CILT: pilot, open-protocol study with two, chronic nonfluent aphasia cases. Restor. Neurol. Neurosci. 32, 483–505. 10.3233/RNN-130365 25015701 PMC4592134

[B86] Medina J. Norise C. Faseyitan O. Coslett H. B. Turkeltaub P. E. Hamilton R. H. (2012). Finding the right words: transcranial magnetic stimulation improves discourse productivity in non-fluent aphasia after stroke. Aphasiology 26, 1153–1168. 10.1080/02687038.2012.710316 23280015 PMC3532848

[B87] Meinzer M. Lindenberg R. Sieg M. M. Nachtigall L. Ulm L. Flöel A. (2014). Transcranial direct current stimulation of the primary motor cortex improves word-retrieval in older adults. Front. Aging Neurosci. 6:253. 10.3389/fnagi.2014.00253 25295004 PMC4172053

[B88] Meinzer M. Mohammadi S. Kugel H. Schiffbauer H. Flöel A. Albers J.. (2010). Integrity of the hippocampus and surrounding white matter is correlated with language training success in aphasia. Neuroimage 53, 283–290. 10.1016/j.neuroimage.2010.06.004 20541018

[B89] Mesulam M. (2008). Primary progressive aphasia pathology. Ann. Neurol. 63, 124–125. 10.1002/ana.20940 16912979

[B90] Mesulam M. M. (2001). Primary progressive aphasia. Ann. Neurol. 49, 425–432. 10.1002/ana.91 11310619

[B91] Mimura M. Kato M. Sano Y. Kojima T. Naeser M. Kashima H. (1998). Prospective and retrospective studies of recovery in aphasia changes in cerebral blood flow and language functions. Brain 121, 2083–2094. 10.1093/brain/121.11.2083 9827768

[B92] Monti A. Cogiamanian F. Marceglia S. Ferrucci R. Mameli F. Mrakic-Sposta S.. (2008). Improved naming after transcranial direct current stimulation in aphasia. J. Neurol. Neurosurg. Psychiatry 79, 451–453. 10.1136/jnnp.2007.135277 18096677

[B93] Moriwaki A. (1991). Polarizing currents increase noradrenaline-elicited accumulation of cyclic AMP in rat cerebral cortex. Brain Res. 544, 248–252. 10.1016/0006-8993(91)90061-Y 1645611

[B94] Mottaghy F. M. Hungs M. Brügmann M. Sparing R. Boroojerdi B. Foltys H.. (1999). Facilitation of picture naming after repetitive transcranial magnetic stimulation. Neurology 53, 1806–1812. 10.1212/WNL.53.8.1806 10563632

[B95] Naeser M. A. Martin P. I. Ho M. Treglia E. Kaplan E. Bashir S.. (2012). Transcranial magnetic stimulation and aphasia rehabilitation. Arch. Phys. Med. Rehabil. 93(1 Suppl.), S26–S34. 10.1016/j.apmr.2011.04.026 22202188 PMC3589757

[B96] Naeser M. A. Martin P. I. Nicholas M. Baker E. H. Seekins H. Helm-Estabrooks N.. (2005). Improved naming after TMS treatments in a chronic, global aphasia patient–case report. Neurocase 11, 182–193. 10.1080/13554790590944663 16006338 PMC1307171

[B97] Naeser M. A. Martin P. I. Theoret H. Kobayashi M. Fregni F. Nicholas M.. (2011). TMS suppression of right pars triangularis, but not pars opercularis, improves naming in aphasia. Brain Lang. 119, 206–213. 10.1016/j.bandl.2011.07.005 21864891 PMC3195843

[B98] National Stroke Association (2008). Available online at: http://www.stroke.org

[B99] Nickels L. (2002). Improving word finding: practices makes (closer to) perfect? Aphasiology 16, 1047–1060. 10.1080/02687040143000618

[B100] Nitsche M. A. Paulus W. (2001). Sustained excitability elevations induced by transcranial DC motor cortex stimulation in humans. Neurology 57, 1899–1901. 10.1212/WNL.57.10.1899 11723286

[B101] Ovadia-Caro S. Villringer K. Fiebach J. Jungehulsing G. J. van der Meer E. Margulies D. S.. (2013). Longitudinal effects of lesions on functional networks after stroke. J. Cereb. Blood Flow Metab. 33, 1279–1285. 10.1038/jcbfm.2013.80 23715061 PMC3734780

[B102] Pascual-Leone A. Tormos J. M. Keenan J. Tarazona F. Cañete C. Catalá M. D. (1998). Study and modulation of human cortical excitability with transcranial magnetic stimulation. J. Clin. Neurophysiol. 15, 333–343. 10.1097/00004691-199807000-00005 9736467

[B103] Pascual-Leone A. Valls-Solé J. Wassermann E. M. Hallett M. (1994). Responses to rapid-rate transcranial magnetic stimulation of the human motor cortex. Brain 117, 847–858. 10.1093/brain/117.4.847 7922470

[B104] Paulus W. (2004). Outlasting excitability shifts induced by direct current stimulation of the human brain. Suppl. Clin. Neurophysiol. 57, 708–714. 10.1016/S1567-424X(09)70411-8 16106673

[B105] Peelle J. E. Troiani V. Gee J. Moore P. McMillan C. Vesely L.. (2008). Sentence comprehension and voxel-based morphometry in progressive nonfluent aphasia, semantic dementia, and nonaphasic frontotemporal dementia. J. Neurolinguist. 21, 418–432. 10.1016/j.jneuroling.2008.01.004 19727332 PMC2598754

[B106] Poeppel D. (2012). The maps problem and the mapping problem: two challenges for a cognitive neuroscience of speech and language. Cogn. Neuropsychol. 29, 34–55. 10.1080/02643294.2012.710600 23017085 PMC3498052

[B107] Price A. R. McAdams H. Grossman M. Hamilton R. H. (2015). A meta-analysis of transcranial direct current stimulation studies examining the reliability of effects on language measures. Brain Stimul. 8, 1093–1100. 10.1016/j.brs.2015.06.013 26210573 PMC4833093

[B108] Prins R. Bastiaanse R. (2006). The early history of aphasiology: from the egyptian surgeons (c.1700 BC) to Broca (1861). Aphasiology 20, 762–791. 10.1080/02687030500399293

[B109] Priori A. Hallett M. Rothwell J. C. (2009). Repetitive transcranial magnetic stimulation or transcranial direct current stimulation? Brain Stimul. 2, 241–245. 10.1016/j.brs.2009.02.004 20633424

[B110] Ren C. L. Zhang G. F. Xia N. Jin C. H. Zhang X. H. Hao J. F.. (2014). Effect of low-frequency rTMS on aphasia in stroke patients: a meta-analysis of randomized controlled trials. PLoS ONE 9:e102557. 10.1371/journal.pone.0102557 25036386 PMC4103829

[B111] Robey R. R. (1994). The efficacy of treatment for aphasic persons: a meta-analysis. Brain Lang. 47, 582–608. 10.1006/brln.1994.1060 7859056

[B112] Robey R. R. Wambaugh J. (1999). Single-subject versus randomized group design. ASHA 41, 14–15. 10570545

[B113] Rogalski E. Cobia D. Harrison T. M. Wieneke C. Thompson C. K. Weintraub S.. (2011). Anatomy of language impairments in primary progressive aphasia. J. Neurosci. 31, 3344–3350. 10.1523/JNEUROSCI.5544-10.2011 21368046 PMC3112000

[B114] Rosen H. J. Petersen S. E. Linenweber M. R. Snyder A. Z. White D. A. Chapman L.. (2000). Neural correlates of recovery from aphasia after damage to left inferior frontal cortex. Neurology 26, 1883–1894. 10.1212/WNL.55.12.1883 11134389

[B115] Rossi S. Hallett M. Rossini P. M. Pascual-Leone A. (2009). Safety, ethical considerations, and application guidelines for the use of transcranial magnetic stimulation in clinical practice and research. Clin. Neurophysiol. 120, 2008–2039. 10.1016/j.clinph.2009.08.016 19833552 PMC3260536

[B116] Rosso C. Perlbarg V. Valabregue R. Arbizu C. Ferrieux S. Alshawan B.. (2014). Broca's area damage is necessary but not sufficient to induce after-effects of cathodal tDCS on the unaffected hemisphere in post-stroke aphasia. Brain Stimul. 7, 627–635. 10.1016/j.brs.2014.06.004 25022472

[B117] Santos M. D. Gagliardi R. J. Mac-Kay A. P. Boggio P. S. Lianza R. Fregni F. (2013). Transcranial direct-current stimulation induced in stroke patients with aphasia: a prospective experimental cohort study. Sao Paulo Med. J. 131, 422–426. 10.1590/1516-3180.2013.1316595 24346782 PMC10871813

[B118] Sarasso S. Santhanam P. Määtta S. Poryazova R. Ferrarelli F. Tononi G.. (2010). Non-fluent aphasia and neural reorganization after speech therapy: insights from human sleep electrophysiology and functional magnetic resonance imaging. Arch. Ital. Biol. 148, 271–278. 21175013 PMC3058764

[B119] Saur D. Hartwigsen G. (2012). Neurobiology of language recovery after stroke: lessons from neuroimaging studies. Arch. Phys. Med. Rehabil. 93, S15–S25. 10.1016/j.apmr.2011.03.036 22202187

[B120] Saur D. Lange R. Baumgaertner A. (2006). Dynamics of language reorganization after stroke. Brain 129, 1371–1384. 10.1093/brain/awl090 16638796

[B121] Schjetnan A. G. Escobar M. L. (2012). *In vivo* BDNF modulation of hippocampal mossy fiber plasticity induced by high frequency stimulation. Hippocampus 22, 1–8. 10.1002/hipo.20866 20848610

[B122] Schlaug G. Marchina S. Norton A. (2009). Evidence for plasticity in white-matter tracts of patients with chronic Broca's aphasia undergoing intense intonation-based speech therapy. Ann. N.Y Acad. Sci. 1169, 385–394. 10.1111/j.1749-6632.2009.04587.x 19673813 PMC2777670

[B123] Shah-Basak P. P. Norise C. Garcia G. Torres J. Faseyitan O. Hamilton R. H. (2015). Individualized treatment with transcranial direct current stimulation in patients with chronic non-fluent aphasia due to stroke. Front. Hum. Neurosci. 9:201. 10.3389/fnhum.2015.00201 25954178 PMC4404833

[B124] Shah-Basak P. P. Wurzman R. Purcell J. B. Gervits F. Hamilton R. (2016). Fields or flows? A comparative metaanalysis of transcranial magnetic and direct current stimulation to treat post-stroke aphasia. Restor. Neurol Neurosci. 34, 537–558. 10.3233/RNN-150616 27163249

[B125] Sharp D. J. Turkheimer F. E. Bose S. K. Scott S. K. Wise R. J. S. (2010). Increased frontoparietal integration after stroke and cognitive recovery. Ann. Neurol. 68, 753–756. 10.1002/ana.21866 20687116

[B126] Shimizu T. Hosaki A. Hino T. Sato M. Komori T. Hirai S.. (2002). Motor cortical disinhibition in the unaffected hemisphere after unilateral cortical stroke. Brain 125, 1896–1907. 10.1093/brain/awf183 12135979

[B127] Strafella A. P. Paus T. Barrett J. Dagher A. (2001). Repetitive transcranial magnetic stimulation of the human prefrontal cortex induces dopamine release in the caudate nucleus. J. Neurosci. 21:RC157. 11459878 10.1523/JNEUROSCI.21-15-j0003.2001 PMC6762641

[B128] Strafella A. P. Paus T. Fraraccio M. Dagher A. (2003). Striatal dopamine release induced by repetitive transcranial magnetic stimulation of the human motor cortex. Brian 126, 2609–2315. 10.1093/brain/awg268 12937078

[B129] Thiel A. Hartmann A. Rubi-Fessen I. Anglade C. Kracht L. Weiduschat N.. (2013). Effects of noninvasive brain stimulation on language networks and recovery in early post-stroke aphasia. Stroke 44, 2240–2246. 10.1161/STROKEAHA.111.000574 23813984

[B130] Thompson H. E. Robson H. Lambon Ralph M. A. Jefferies E. (2015). Varieties of semantic ‘access’ deficit in Wernicke's aphasia and semantic aphasia. Brain 138, 3776–3792. 10.1093/brain/awv281 26454668 PMC4655340

[B131] Töpper R. Mottaghy F. M. Brügmann M. Noth J. Huber W. (1998). Facilitation of picture naming by focal transcranial magnetic stimulation of Wernicke's area. Exp. Brain Res. 121, 371–378. 10.1007/s002210050471 9746143

[B132] Trebbastoni A. Raccah R. de Lena C. Zangen A. Inghilleri M. (2013). Repetitive deep transcranial magnetic stimulation improves verbal fluency and written language in a patient with primary progressive aphasia-logopenic variant (LPPA). Brain Stimul. 6, 545–553. 10.1016/j.brs.2012.09.014 23122915

[B133] Tsapkini K. Frangakis C. Gomez Y. Davis C. Hillis A. E. (2014). Augmentation of spelling therapy with transcranial direct current stimulation in primary progressive aphasia: preliminary results and challenges. Aphasiology 28, 1112–1130. 10.1080/02687038.2014.930410 26097278 PMC4470615

[B134] Turkeltaub P. E. (2015). Brain stimulation and the role of the right hemisphere in aphasia recovery. Curr. Neurol. Neurosci. Rep. 15:72. 10.1007/s11910-015-0593-6 26396038

[B135] Turkeltaub P. E. Coslett H. B. Thomas A. L. Faseyitan O. Benson J. Norise C.. (2012). The right hemisphere is not unitary in its role in aphasia recovery. Cortex 48, 1179–1186. 10.1016/j.cortex.2011.06.010 21794852 PMC3221765

[B136] Turkeltaub P. E. Eickhoff S. B. Laird A. R. Fox M. Wiener M. Fox P. (2011a). Minimizing within-experiment and within-group effects in activation likelihood estimation meta-analyses. Hum. Brain Mapp. 33, 1–13. 10.1002/hbm.21186 21305667 PMC4791073

[B137] Turkeltaub P. E. Messing S. Norise C. Hamilton R. H. (2011b). Are networks for residual language function and recovery consistent across aphasic patients? Neurology 76, 1726–1734. 10.1212/WNL.0b013e31821a44c1 21576689 PMC3100133

[B138] Vandenbulcke M. Peeters R. Van Hecke P. Vandenberghe R. (2005). Anterior temporal laterality in primary progressive aphasia shifts to the right. Ann. Neurol. 58, 362–370. 10.1002/ana.20588 16130090

[B139] van Hees S. McMahon K. Angwin A. de Zubicaray G. Read S. Copland D. A. (2014). A functional MRI study of the relationship between naming treatment outcomes and resting state functional connectivity in post-stroke aphasia. Hum. Brain Mapp. 35, 3919–3931. 10.1002/hbm.22448 24453137 PMC6869730

[B140] Vestito L. Rosellini S. Mantero M. Bandini F. (2014). Long-term effects of transcranial direct-current stimulation in chronic post-stroke aphasia: a pilot study. Front. Hum. Neurosci. 8:785. 10.3389/fnhum.2014.00785 25352798 PMC4196539

[B141] Vines B. W. Norton A. C. Schlaug G. (2011). Non-invasive brain stimulation enhances the effects of melodic intonation therapy. Front. Psychol. 2:230. 10.3389/fpsyg.2011.00230 21980313 PMC3180169

[B142] Vuksanović J. Jelić M. B. Milanović S. D. Kačar K. Konstantinović L. Filipović S. R. (2015). Improvement of language functions in a chronic non-fluent post-stroke aphasic patient following bilateral sequential theta burst magnetic stimulation. Neurocase 21, 244–250. 10.1080/13554794.2014.890731 24579976

[B143] Wade D. T. Hewer R. L. David R. M. Enderby P. M. (1986). Aphasia after stroke: natural history and associated deficits. J. Neurol. Neurosurg. Psychiatry 49, 11–16. 10.1136/jnnp.49.1.11 2420939 PMC1028640

[B144] Waldowski K. Seniów J. Leśniak M. Iwański S. Członkowska A. (2012). Effect of low-frequency repetitive transcranial magnetic stimulation on naming abilities in early-stroke aphasic patients: a prospective, randomized, double-blind sham-controlled study. Sci. World J. 2012:518568. 10.1100/2012/518568 23213288 PMC3508571

[B145] Wang J. Wu D. Chen Y. Yuan Y. Zhang M. (2013). Effects of transcranial direct current stimulation on language improvement and cortical activation in nonfluent variant primary progressive aphasia. Neurosci. Lett. 549, 29–33. 10.1016/j.neulet.2013.06.019 23800543

[B146] Warburton E. Price C. J. Swinburn K. Wise R. J. (1999). Mechanisms of recovery from aphasia: evidence from positron emission tomography studies. J. Neurol. Neurosurg. Psychiatry 66, 155–161. 10.1136/jnnp.66.2.155 10071093 PMC1736204

[B147] Weiduschat N. Thiel A. Rubi-Fessen I. Hartmann A. Kessler J. Merl P.. (2011). Effects of repetitive transcranial magnetic stimulation in aphasic stroke: a randomized controlled pilot study. Stroke 42, 409–415. 10.1161/STROKEAHA.110.597864 21164121

[B148] Wernicke C. (1874). Der Aphasische Symptomenkomplex. Berlin: Fischer.

[B149] Wilson S. M. Dronkers N. F. Ogar J. M. Jang J. Growdon M. E. Agosta F.. (2010). Neural correlates of syntactic processing in the nonfluent variant of primary progressive aphasia. J. Neurosci. 30, 16845–16854. 10.1523/JNEUROSCI.2547-10.2010 21159955 PMC3024013

[B150] Wilson S. M. Galantucci S. Tartaglia M. C. Rising K. Patterson D. K. Henry M. L.. (2011). Syntactic processing depends on dorsal language tracts. Neuron 72, 397–403. 10.1016/j.neuron.2011.09.014 22017996 PMC3201770

[B151] Winhuisen L. Thiel A. Schumacher B. Kessler J. Rudolf J. Haupt W. F.. (2005). Role of the contralateral inferior frontal gyrus in recovery of language function in post-stroke aphasia: a combined repetitive transcranial magnetic stimulation and positron emission tomography study. Stroke 36, 1759–1763. 10.1161/01.STR.0000174487.81126.ef 16020770

[B152] Wu D. Wang J. Yuan Y. (2015). Effects of transcranial direct current stimulation on naming and cortical excitability in stroke patients with aphasia. Neurosci. Lett. 589, 115–120. 10.1016/j.neulet.2015.01.045 25603474

[B153] Xing S. Lacey E. H. Skipper-Kallal L. M. Jiang X. Harris-Love M. L. Zeng J.. (2016). Right hemisphere grey matter structure and language outcomes in chronic left hemisphere stroke. Brain 139, 227–241. 10.1093/brain/awv323 26521078 PMC4990653

[B154] Yang M. Li J. Li Y. Li R. Pang Y. Yao D.. (2016). Altered intrinsic regional activity and interregional functional connectivity in post-stroke aphasia. Sci. Rep. 6:24803. 10.1038/srep24803 27091494 PMC4835729

[B155] You D. S. Kim D. Y. Chun M. H. Jung S. E. Park S. J. (2011). Cathodal transcranial direct current stimulation of the right Wernicke's area improves comprehension in subacute stroke patients. Brain Lang. 119, 1–5. 10.1016/j.bandl.2011.05.002 21641021

[B156] Yourganov G. Smith K. G. Fridriksson J. Rorden C. (2015). Predicting aphasia type from brain damage measured with structural MRI. Cortex 73, 203–215. 10.1016/j.cortex.2015.09.005 26465238 PMC4689665

